# Synovial sarcoma of the maxillary sinus – A rare condition managed with a rationalized surgery

**DOI:** 10.1016/j.amsu.2021.102538

**Published:** 2021-07-03

**Authors:** Burhan Hannoun, Ibrahim Hannoun, Albaraa Bara, Areej Alassaf, Eyad M. Chatty

**Affiliations:** a Faculty of Dentistry, Damascus University, Damascus, Syrian Arab Republic; b Faculty of Medicine, Damascus University, Syrian Arab Republic; c Al-Mouassat University Hospital, Syria; d Syrian College of Pathologists, Department of Pathology, Alassad University Hospital, Damascus, Syria; e Department of Pathology, Mowasat University Hospital, Damascus, Syria; f Board of Trustees, Kalamoon University, Damascus, Syria

**Keywords:** Synovial sarcoma, Maxilla, Pterygopalatine, Head and neck, Case report

## Abstract

**Introduction:**

and importance: Synovial sarcoma is a type of spindle cell tumors with unknown cellular origin. It can present anywhere throughout the body; however, its onset in the maxillary sinus is an extremely rare condition, making it hard to diagnose. This tumor occurs equally, without predilection for males or females, and the incidence peaks in the age of 35. The diagnosis is confirmed by histopathological study, and the main treatment is complete surgical excision.

**Case presentation:**

We are reporting a case of a 53-year-old male with a left sided hearing loss accompanied by a left sided nasal block and a vague facial and dental pain.

**Clinical discussion:**

Magnetic Resonance Imaging (MRI) showed a heterogeneously enhancing tumor in the maxillary sinus that extended to the pterygopalatine fossa and other surrounding structures, and a biopsy showed the tumor to be a synovial sarcoma. The tumor was managed with a less aggressive curative surgery, and was put on an adjuvant radiotherapy, and is being followed regularly; with no recurrence 5 months after therapy.

**Conclusion:**

In conclusion, we are writing this report to introduce a case of synovial sarcoma in a rare location that was managed by a “cosmetically oriented” curative surgery, satisfactory results and prognosis.

## Highlights

• Synovial sarcoma is a rare type of spindle cell tumors.
• Synovial sarcoma of the maxillary sinus is a quite rare condition.
• The term “synovial sarcoma” is an old misnomer.
• The cellular origin of Synovial sarcoma is still unknown.
• The main treatment for synovial sarcoma is surgical excision.

## 1 Introduction

Synovial sarcoma is a type of spindle cell tumors which represents 10% of soft tissue sarcomas. The term “synovial sarcoma” was used in early literature due to the microscopic appearance of this sarcoma that is similar to a developing synovium, however, the origin of this tumor is still unknown [1].

Although synovial sarcoma can arise in various sites throughout the body [2], its onset in the maxillary sinus is an extremely rare condition, making it hard to diagnose, especially when the tumor is small, where it can grow slowly and manifests with nonspecific symptoms [3]. In this paper, we report an extremely rare case of synovial sarcoma in the maxillary region involving the adjacent structures that was managed with a “cosmetically oriented” curative surgery. This work has been reported in line with the SCARE criteria [4].

## 2 Case presentation

**Fig. 1:**
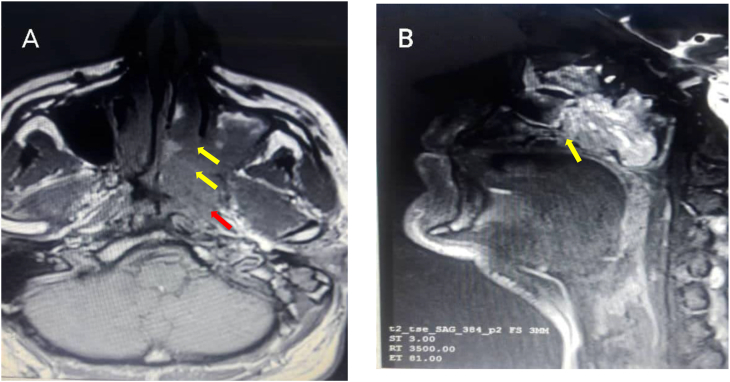
MR images showing a heterogeneously enhancing tumor in the maxillary sinus. A: the tumor eroded the medial wall of the sinus (yellow arrows) and reached the pterygopalatine fossa (red arrow). B: the tumor eroded the posterior aspect of the hard palate (yellow arrow). (For interpretation of the references to colour in this figure legend, the reader is referred to the Web version of this article.)

A 53-year-old male complained of a left sided hearing loss for few months as well as a left sided nasal block, then developed a vague facial and dental pain few weeks prior to his visit. Physical examination showed the hearing loss to be of conductive nature, reduced sensation in the area of skin innervated by the infraorbital nerve, and no lymphadenopathy was noted. MRI showed a heterogeneously enhancing tumor measuring 38 mm × 28 mm × 42 mm in the maxillary sinus that eroded the medial wall of the sinus, extended to the posterior aspect of the nasal cavity eroding the posterior aspect of the hard palate, and extended posteriorly thereafter closing the pharyngeal aspect of the Eustachian tube causing otitis media with effusion, and reaching the pterygopalatine fossa eventually (Fig. 1). No signs of metastasis were found.

**Fig. 2:**
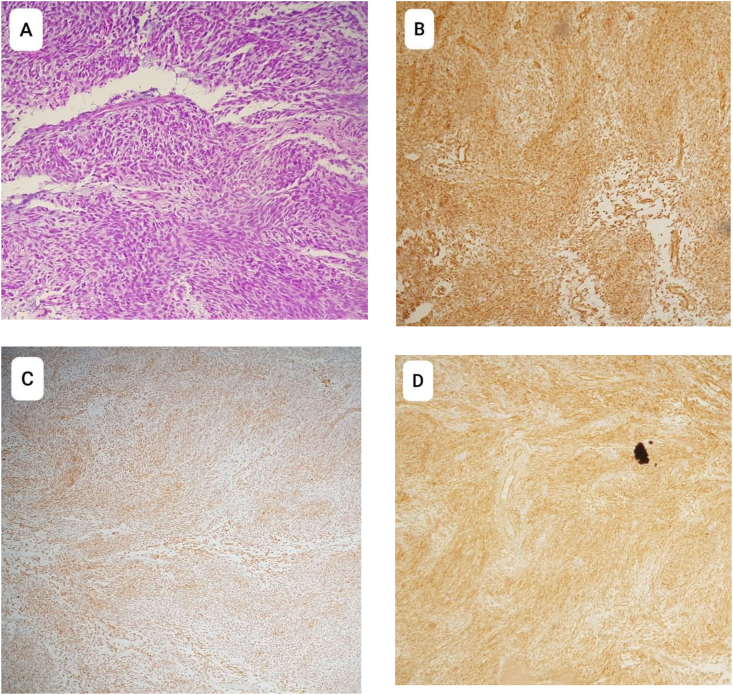
Microscopic examination and immunohistochemical study. A: Hematoxylin and eosin staining showing a monophasic spindle cell sarcoma. B: positive Vimentin staining. C: positive Bcl-2 staining. D: positive CD99 staining.

**Fig. 3:**
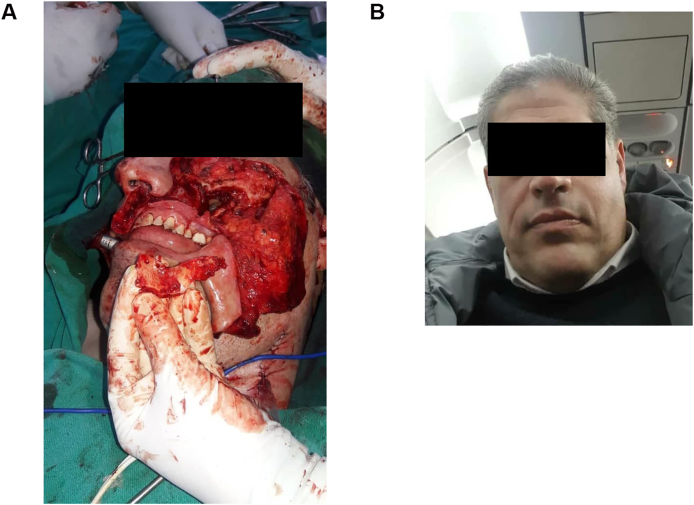
The cosmetic result after the surgery. A: an anonymized clinical figure during the surgery. B: an anonymized clinical figure after the surgery.

All information was pointing towards a possible malignant tumor; Therefore, a biopsy was endoscopically obtained from the tumor in the maxillary sinus. Microscopic examination of the biopsy showed small ovoid to spindle-shaped cells with scanty cytoplasm, some of which demonstrated signs of atypical mitosis. The cells were arranged in short interlacing sheets, with no epithelial components to be found; all of which pointed towards a monophasic spindle cell tumor. Immunohistochemically, the tumor was positive for Vimentin, Bcl-2, and CD99 (Fig. 2), and negative for CK, with a Ki67 proliferative index of 3%; Thus, a monophasic spindle cell sarcoma was diagnosed. A TNM classification of T4aN0M0 was put. Surgery was chosen as the treatment of choice. Under general anesthesia, the coronoid process of the mandible was removed to create a window through which the posterior aspect of the tumor could be reached, the patient then underwent partial left maxillectomy in which the alveolar arch (up to the 1st premolar) was preserved, the left pterygoid processes of the sphenoid bone were also excised, and the tumor was resected en bloc; the anterior wall of the maxillary bone was separated and preserved with plans to reattach it should the surgical margins be clear; which were proven so on histological examination of the frozen section. The patient was put on an adjuvant radiotherapy, and is being followed on a monthly basis for the first year, and yearly thereafter; with no recurrence 5 months after therapy. The cosmetic result after the surgery was good (Fig. 3).

## 3 Discussion

Although synovial sarcoma is considered a quite common soft tissue tumor in the limbs, it can present anywhere in the body with the maxillary sinus being an extremely rare location [5]. The term “synovial sarcoma” is a misnomer designated by Knox in 1936 because of its histologic resemblance of synovial tissues; However, the cellular origin of the tumor is still unknown, with some studies suggesting an epithelial origin [1], while others suggesting the myoblasts as the original cells [6]. Synovial sarcoma is considered to be a high grade sarcoma by definition, it occurs equally without predilection for males or females, and the incidence peaks in the age of 35 [1]. Diagnosing synovial sarcoma when it presents in its uncommon locations is a challenging process, however, the presence of some clinical indicators such as the relatively rapid progression of symptoms, as well as “disrespecting the anatomical distribution” should direct the physician's attention towards the possible malignant nature of the disease. Imaging studies also help determining the nature of the defect, as MRI can show a heterogeneously enhancing lesion with invasion of the surrounding structures; However, microscopic and immunohistochemical examination remain the definitive diagnostic tools [1]. The prognosis of synovial sarcoma is affected by many factors such as the tumor size, marginal clarity, mitotic activity, neurovascular invasion, and Ki67 proliferative index [7]. Some studies have also shown a better prognosis for head and neck synovial sarcoma when compared with synovial sarcoma of the extremities [8]; which all should be kept in mind when deciding the course of management. Surgical treatment is the main approach when dealing with synovial sarcoma; with “en bloc” resection being the primary surgical principle used in such cases, taking special care in trying to achieve a clear border resection if possible. This approach, however, tends to leave a significant cosmetic and functional defects should the surgeon be overzealous with marginal excision width, especially in regions of complex anatomy such as the head and neck region. Adjuvant radiotherapy is usually recommended.

## 4 Conclusion

We are reporting a case of maxillary sinus synovial sarcoma that was managed with partial maxillectomy with alveolar arch and anterior sinus wall preservation after most prognostic factors were found to be on the safe side, and the surgical margins were clear on microscopic examination; with satisfactory prognosis and a 5-months disease free period.

## Declaration of competing interest

All the authors declare that they have no conflicts of interest.

## Registration of research studies

N/A.
